# Derivates of the Antifungal Peptide Cm-p5 Inhibit Development of *Candida auris* Biofilms In Vitro

**DOI:** 10.3390/antibiotics9070363

**Published:** 2020-06-27

**Authors:** Dennis Kubiczek, Heinz Raber, Melaine Gonzalez-García, Fidel Morales-Vicente, Ludger Staendker, Anselmo J. Otero-Gonzalez, Frank Rosenau

**Affiliations:** 1 Institute of Pharmaceutical Biotechnology, Ulm University, 89081 Ulm, Germany; dennis.kubiczek@uni-ulm.de (D.K.); Heinz.Raber@uni-ulm.de (H.R.); 2 Center for Protein Studies, Faculty of Biology, University of Havana, 25 Str. and I Str., La Habana 10400, Cuba; mgonzalez@fbio.uh.cu (M.G.-G.); aotero@fbio.uh.cu (A.J.O.-G.); 3 General Chemistry Department, Faculty of Chemistry, University of Havana, Zapata y G, La Habana 10400, Cuba; femvicente@gmail.com; 4 Synthetic Peptides Group, Center for Genetic Engineering and Biotechnology, 10600 La Habana, Cuba; 5 Core Facility for Functional Peptidomics, Ulm Peptide Pharmaceuticals (U-PEP), Faculty of Medicine, Ulm University, 89081 Ulm, Germany; ludger.staendker@uni-ulm.de

**Keywords:** antimicrobial peptide, *Candida auris*, *Candida albicans*, *Candida parapsilosis*, peptide drug

## Abstract

Growth in biofilms as a fascinating and complex microbial lifestyle has become widely accepted as one of the key features of pathogenic microbes, to successfully express their full virulence potential and environmental persistence. This also increases the threat posed by *Candida auris*, which has a high intrinsic ability to persist on abiotic surfaces including those of surgical instruments and medical tubing. In a previous study, cyclic and helical-stabilized analogues of the antifungal peptide Cm-p5 were designed and synthetized, and proved to have increased activities against *C.* *albicans* and *C. parapsilosis*, but not against planktonic *C. auris* cells cultivated in suspension cultures. Here, we demonstrate, initially, that these derivatives, however, exhibited semi-inhibitory concentrations between 10–21 µg/mL toward *C. auris* biofilms. Maturated biofilms were also arrested between 71–97%. These novel biofilm inhibitors may open urgently needed new routes for the development of novel drugs and treatments for the next stage of fight against *C. auris*.

## 1. Introduction

Compared to microbiological cultivation in laboratories, biofilms formed on biotic or abiotic surfaces are the most relevant and the “normal” lifestyle for microorganisms in general [1]. This is also true for important pathogenic bacteria and lower eukaryotes. It has been estimated that biofilms are associated with 65% of nosocomial infections or up to over 80% for all microbial infections [2,3] (as estimated by the NIH [4]). *Saccharomycetaceae* of the genus *Candida* represent a class of highly important pathogens with *C. albicans*, *C. parapsilosis* and *C. glabrata* being among the most abundant species in clinical fungal infections [5]. *Candida auris*, however, is a relatively new pathogen that was first isolated in 2009 from a Japanese patient [6]. It causes severe invasive infections in hospitalized patients, which lead to high mortality rates between 35 and 60% [7,8]. A special threat is that strains of *C. auris* with multiple resistances against commonly used antifungal drugs have been reported to occur independently in different countries/continents worldwide [9]. Thus, both the U.S. Center for Disease Control (CDC) and the European Centre for Disease Prevention and Control (ECDC) have released clinical alerts, initiating also a broad public discourse, identifying *C. auris* as an emerging “superbug” [10]. Multi-resistance of *C. auris* against classical antifungal drugs like fluconazole or amphotericin B has been discussed to be caused by the activity of ABC-type major facilitator superfamily (MFS) efflux pumps [11], which are even upregulated in *C. auris* biofilms [12]. The resistance against classical antifungal drugs has provoked studies to identify novel compounds, which potentially avoid the impact of efflux mechanisms, due to alternative modes of action. One promising class of therapeutic molecules are antimicrobial peptides (AMPs).

**Figure 1:**
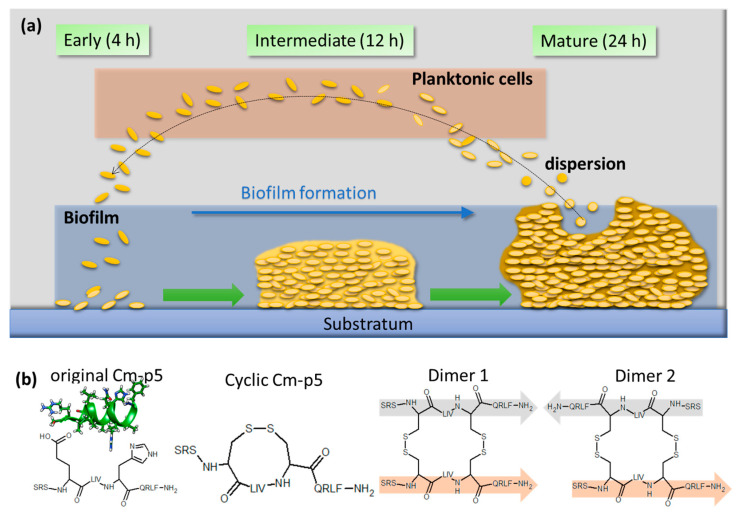
Schematic overview of *C. auris* biofilm development and the structure of the antifungal Cm-p5 peptide and its derivatives. (**a**) Classification of the three major steps in biofilm formation of *C. auris*. The early phase (4 h) is characterized by the adhesion of planktonic cells to the substratum. Proliferation and extracellular matrix production take place in the intermediate phase (12 h). The mature biofilm features the production of virulence factors and dispersion of planktonic cells from the biofilm (24 h). (**b**) Structure of the original Cm-p5 and its helical stabilized derivatives. NMR structure shown for the original helical Cm-p5 peptide can be accessed in the RSCB protein data bank (PDB: 2MP9).

Most AMPs simply have a physical mode of action, in a way that they reduce the functional integrity of microbial cell walls by pore formation or related activities [13,14], and can be considered to provide excellent treatment options even against organisms resistant to conventional antifungal drugs. The AMP Cm-p5 derived from a natural peptide originally isolated from the coastal mollusk *Cenchritis muricatus* exhibited efficient antifungal activity against different fungal pathogens [15] including *C. auris* [16,17], with only marginal cytotoxicity towards mammalian cells [15]. Therefore, Cm-p5 provides an attractive therapeutic index, opening various opportunities for developing efficient and safe antifungal drugs, and has been used in so-called smart wound dressings as a first line of defense against infections caused by *C. auris* contaminated wounds [16]. In a rational design study cyclic and helical-stabilized analogues of Cm-p5 were synthetized and proved increased activities against *C. albicans* and *C. parapsilosis*, but not against planktonic *C. auris* cells cultivated in suspension cultures. Here we demonstrate that these derivatives, however, exhibit a considerable activity towards *C. auris* biofilms formed on an abiotic substratum, inhibiting the growth of mature biofilm. We believe that this novel activity of these synthetic Cm-p5 derivatives may provide new avenues towards the development of novel anti *C. auris* biofilm treatment options. Since the early days of biofilm research, this fascinating and complex microbial lifestyle has become widely accepted as one of the key features of pathogenic microbes to also successfully express their full virulence potential and environmental persistence [3,12,18]. This is also true not only in the case of *C. auris*, but gains an even higher impact by the fact that *C. auris* has a remarkably higher intrinsic ability to persist on abiotic surfaces [19,20], probably including those of surgical instruments and medical tubing. Traditionally the formation of biofilms is a central process in microbial development, and it is divided into a series of distinct developmental steps [21]. For *C. auris*, three major phases of biofilm growth have been defined [12]. The first phase is characterized by adhesion of planktonic cells (potentially released from dissolved biofilms) to the substratum, and occurs within the early phase of the first 4 h, whereas the second or intermediate phase is characterized by proliferation of the immobilized cells, enhancement of biosynthesis of components for the extra cellular matrix (ECM) and, as a major physiological adaptation, the upregulation of efflux pumps as molecular key machineries to develop multi-resistance. The following maturation to elaborate biofilm after 24 h involves the completion of ECM synthesis and the start of virulence factor production, and is denoted as phase three [12] (Figure 1a).

## 2. Results and Discussion

The original Cm-p5 peptide and the lead structure for the derivatives discussed here is a 12 mer with helical 3D-structure as determined by NMR [15] (Figure 1b). Efforts to optimize its antifungal activity delivered three rationally designed helically stabilized derivatives. By the introduction of cysteine residues and oxidation, intramolecular cyclisation was achieved (Figure 1b, cyclic), or the modified monomers were used to generate parallel and anti-parallel dimers by intermolecular disulfide bond formation, respectively (Figure 1b, dimer 1, dimer 2) [17].

**Figure 2:**
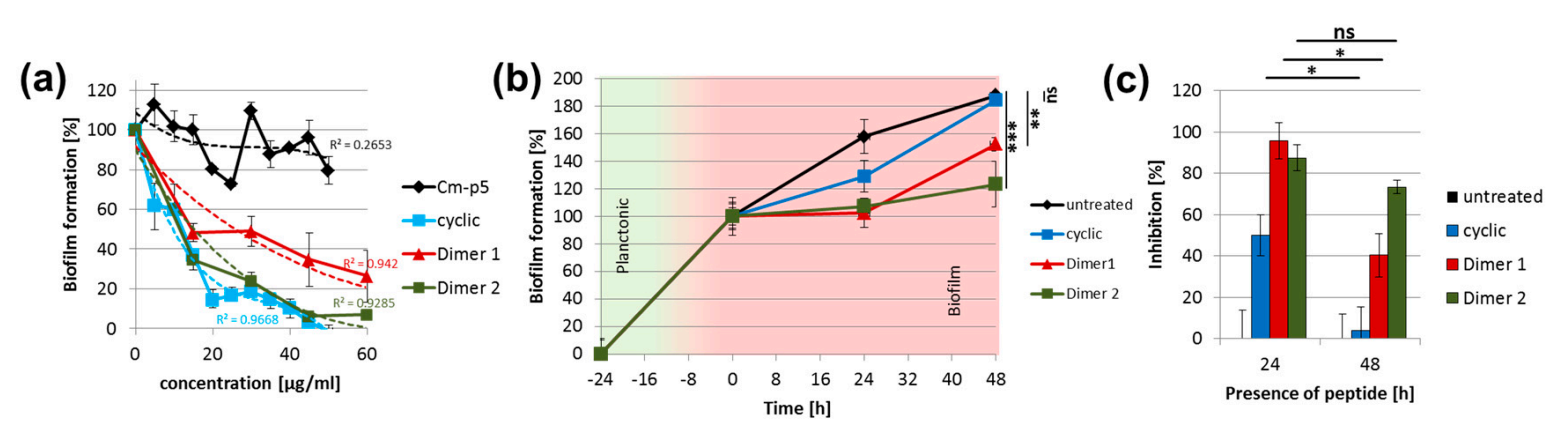
Biofilm inhibition by Cm-p5 derivatives. (**a**) Dose-dependent inhibition of *C. auris* de novo biofilm formation by cyclic Cm-p5, dimer 1 and dimer 2 quantified with crystal violet after 24 h. Inhibitors were present throughout the growth. R^2^ represents the coefficient of determination of the applied trendlines. (**b**) Arrest of biofilm growth by Cm-p5 derivatives added to mature biofilms (at t = 0 and 24 h). (**c**) Inhibition calculated from (**b**) relative to the mature biofilm (t = 0 h). A constant biofilm biomass thereby depicts an inhibition of 100%. All experiments were performed in triplicate, and statistical analysis by a t-test was performed where applicable. *p* values < 0.05 were considered significant. * denotes *p* < 0.05, ** < 0.01, *** < 0.001.

The cyclic derivative showed the desired higher activity against *C. parapsilosis* and *C. albicans*, whereas the efficacy of the dimers was rather moderate. Against planktonic cells of *C. auris*, the efficacy of all derivatives was significantly diminished [17]. However, when the derivatives were tested in an experimental setup, in which the cells were allowed to form biofilms on the polystyrene surface of microtiter plates in the presence of increasing concentrations of Cm-p5 as a control and the peptide derivatives, the latter ones showed anti-biofilm activity, and were more effective than the original Cm-p5 (Figure 2a). With untreated cells forming the reference biofilm, all peptides showed inhibitory effects in a dose dependent fashion. Whereas the activity of Cm-p5 was rather limited, the cyclic peptide and the two dimers showed semi-inhibitory concentrations in this biofilm assay between 10–21 µg/mL. Interestingly, these concentrations were below the minimal inhibitory concentration determined for growth inhibition of *C. auris* cells in submersed cultures [17] (Figure 2a). This suggested that the derivative peptides, in contrast to the unmodified original Cm-p5, may have distinct additional activities on biofilm cells, which may be mechanistically different from their probable biocidal membrane disruptive effects on the cell walls of *C. auris* planktonic cells. However, at this point of the study, it was not clear whether this probable new biofilm dissolving activity, which was observed during growth and development of biofilms in the presence of these peptides, can also act when they were applied to already matured biofilms to limit further growth of the biofilm biomass. When biofilms were allowed to form to the end of phase one (24 h), and were then treated with the cyclic peptide or the two dimers, the development was quantitatively arrested for dimer 1 and dimer 2, with 95% and 87% inhibition of further growth, respectively, and considerably retarded to 49% inhibition for the cyclic peptide (Figure 2b,c). For the cyclic peptide and dimer 1, the inhibitory effect lost its clarity after additional 24 h (48 h, Figure 2c), suggesting a resumption of biofilm development. However, dimer 2 maintained its inhibitory effect in this extended period of time (Figure 2c). The observed differences between the three derivatives concerning their biofilm inhibition capability in this type of assay may not exclusively be the result from their postulated intrinsic activity in addition to their membrane disruptive properties as AMPs, but may also be a consequence of different individual susceptibilities of these peptides towards proteolytic degradation in the biofilm. This idea is supported by the findings of Kean et al., which show that, in mature biofilms with candidapepsin-5 a prominent, virulence associated protease/peptidase is upregulated [12], which has multiple roles for *Candida spec.* in epithelial invasion [22], and probably the consolidation of elaborate mature biofilms [23]. This enzyme belongs to global biofilm-specific proteolytic activities, which may also address the antifungal peptides. This biofilm specificity, in turn, may explain why inhibition worked in the experiments presented in this study, when the peptides were present already during the early adhesion phase, before biofilm specific responses in the physiology of *C. auris* could occur. Although the exact dimension of proteolytic degradation of the peptides by *C. auris* biofilms needs to be determined in detail, it is reasonable to speculate that novel variants of the cyclic peptide and dimer 1 and 2 modified by acetylation to increase their resilience against protease activities may be a beneficial next step to further improve these inhibitors. The inhibitory effects of the cyclic Cm-p5 variant and the two dimers on early stage biofilms were unexpectedly pronounced and positive, compared to the original unmodified Cm-p5, especially with respect to their extremely limited activity towards planktonic cells. This may in fact qualify them as interesting molecules with a novel mode of action and specificity towards biofilms and, thus, as valuable targets for future in-depth studies to elucidate their properties and working mechanism. They may also open urgently needed new routes for the development of novel drugs and treatments for the next stage of fight against *C. auris*, as mankind is encountering, for the first time, a multidrug resistant pathogenic yeast with an exceptionally high capacity to cause clusters of invasive infections in medical centers around the world.

## 3. Materials and Methods

Yeast extract, peptone, glucose, crystal violet and glacial acetic acid were obtained from Carl Roth GmbH (Karlsruhe, Germany), RPMI-1640 medium supplemented with L-glutamine was purchased at Thermo Fisher Scientific (Waltham, MA, USA). Statistical analysis was performed by two tailed unpaired student t-tests, where applicable. *p* values < 0.05 were considered significant. * denotes *p* < 0.05, ** < 0.01, *** < 0.001.

### 3.1. Cultivation of C. auris

*Candida auris* was purchased from DSMZ (DSMZ-No. 21092) and grown on YPD Agar (1% *w*/*v* yeast extract, 2% *w*/*v* peptone, 2% *w*/*v* glucose, 1.5% Agar). For suspension cultures, 10 mL YPD medium in a 100 mL Erlenmayer flask was inoculated with a single colony and grown at 37 °C and orbital shaking at 150 rpm.

### 3.2. Biofilm Formation and Quantification

Biofilms were basically formed and analyzed, as described previously [20,24,25] in triplicate. In brief, 2.5 × 10^3^ yeast cells were seeded in 200 µL RPMI-1640 medium supplemented with L-glutamine in a flat bottomed, 96-well polystyrene microtiter plates (Sarstedt AG & Co. KG, Nümbrecht, Germany), and incubated at 37 °C without agitation for 24 h. The effect of the different Cm-p5 derivates on the biofilm formation was tested in the presence of Cm-p5 or its derivatives at different concentrations. The biofilm was quantified by a crystal violet assay, which was originally developed for bacteria by George O’ Toole [21,25], and is also widely used for *Candida* biofilms [20,24,26,27]. Planktonic cells were removed with the supernatant and the mature biofilms were washed twice with 200 µL water. Subsequently, biofilms were stained with 200 µL of a 0.1% (*w*/*v*) crystal violet solution for 15 min. The supernatant was removed and the biofilms were washed twice with 200 µL water to get rid of excess crystal violet. The stained biofilms were air dried for 24 h at 25 °C, and finally destained using 200 µL of 30% acetic acid (15 min, 25 °C). The supernatant was transferred to a fresh 96 well plate and the absorbance at 570 nm was measured using a Tecan infinite M200 microplate reader to quantify the biofilm biomass.

### 3.3. Effects of Cm-p5 Derivatives on Mature Biofilms

*C. auris* biofilms were grown in triplicate, as described above. After 24 h the supernatant was removed from the mature biofilm, and the medium was replaced with 200 µL RPMI-1649 containing 15 µg/mL of cyclic Cm-p5, dimer 1 or dimer 2. This was repeated after further 24 h. The biofilm was quantified as described above at each individual time point. For each time point, three individual biofilms, grown in parallel, were quantified for each peptide. The biofilm inhibition was calculated with the equation:$\;Inhibition=100-\left(\frac{\;\Delta biomass\;treated\;(\Delta absorbance\;\left(t_{24h/48h}-t_{0}\right)}{\;\Delta biomass\;untreated\;(\Delta absorbance\;\left(t_{24h/48h}-t_{0}\right)}*100\right)$. with *t*_0_ = time point at 24 h growth; *t*_24h/48h_ = time point at 24 h or 48 h after treatment.

## 4. Conclusions

We have demonstrated that variants of the already described antimicrobial peptide Cm-p5 can be used to inhibit de novo biofilm formation by the pathogenic yeast *C. auris*. The maturation of growing biofilms can be stopped and the development of additional biomass in these biofilms is arrested upon addition of the peptide inhibitors.

## References

[1] Nadell C.D., Bassler B.L., Levin S.A. (2008). Observing bacteria through the lens of social evolution. J. Biol..

[2] Licking E. (1999). Getting a grip on bacterial slime. Bus. Week.

[3] Davies D. (2003). Understanding biofilm resistance to antibacterial agents. Nat. Rev. Drug Discov..

[4] Fox E.P., Nobile C.J. (2012). A sticky situation: Untangling the transcriptional network controlling biofilm development in Candida albicans. Transcription.

[5] Papon N., Courdavault V., Clastre M., Bennett R.J. (2013). Emerging and Emerged Pathogenic Candida Species: Beyond the Candida albicans Paradigm. PLoS Pathog..

[6] Satoh K., Makimura K., Hasumi Y., Nishiyama Y., Uchida K., Yamaguchi H. (2009). Candida auris sp. nov., a novel ascomycetous yeast isolated from the external ear canal of an inpatient in a Japanese hospital. Microbiol. Immunol..

[7] Morales-López S.E., Parra-Giraldo C.M., Ceballos-Garzón A., Martínez H.P., Rodríguez G.J., Álvarez-Moreno C.A., Rodríguez J.Y. (2017). Invasive infections with multidrug-resistant yeast Candida auris, Colombia. Emerg. Infect. Dis..

[8] Lockhart S.R., Etienne K.A., Vallabhaneni S., Farooqi J., Chowdhary A., Govender N.P., Colombo A.L., Calvo B., Cuomo C.A., Desjardins C.A. (2017). Simultaneous emergence of multidrug-resistant candida auris on 3 continents confirmed by whole-genome sequencing and epidemiological analyses. Clin. Infect. Dis..

[9] Osei Sekyere J. (2018). Candida auris: A systematic review and meta-analysis of current updates on an emerging multidrug-resistant pathogen. Microbiologyopen.

[10] Forbes. https://www.forbes.com/sites/judystone/2017/08/24/candida-auris-a-new-fungal-superbug-emerging-as-a-global-threat/.

[11] Ben-Ami R., Berman J., Novikov A., Bash E., Shachor-Meyouhas Y., Zakin S., Maor Y., Tarabia J., Schechner V., Adler A. (2017). Multidrug-resistant candida haemulonii and C. Auris, tel aviv, Israel. Emerg. Infect. Dis..

[12] Kean R., Delaney C., Sherry L., Borman A., Johnson E.M., Richardson M.D., Rautemaa-Richardson R., Williams C., Ramage G. (2018). Transcriptome Assembly and Profiling of Candida auris Reveals Novel Insights into Biofilm-Mediated Resistance. mSphere.

[13] Ciociola T., Giovati L., Conti S., Magliani W., Santinoli C., Polonelli L. (2016). Natural and synthetic peptides with antifungal activity. Future Med. Chem..

[14] Rautenbach M., Troskie A.M., Vosloo J.A. (2016). Antifungal peptides: To be or not to be membrane active. Biochimie.

[15] López-Abarrategui C., McBeth C., Mandal S.M., Sun Z.J., Heffron G., Alba-Menéndez A., Migliolo L., Reyes-Acosta O., García-Villarino M., Nolasco D.O. (2015). Cm-p5: An antifungal hydrophilic peptide derived from the coastal mollusk Cenchritis muricatus (Gastropoda: Littorinidae). FASEB J..

[16] Kubiczek D., Flaig C., Raber H., Dietz S., Kissmann A.K., Heerde T., Bodenberger N., Wittgens A., González-Garcia M., Kang F. (2020). A Cerberus-Inspired Anti-Infective Multicomponent Gatekeeper Hydrogel against Infections with the Emerging “Superbug” Yeast Candida auris. Macromol. Biosci..

[17] Morales-Vicente F.E., González-Garcia M., Diaz Pico E., Moreno-Castillo E., Garay H.E., Rosi P.E., Jimenez A.M., Campos-Delgado J.A., Rivera D.G., Chinea G. (2019). Design of a Helical-Stabilized, Cyclic, and Nontoxic Analogue of the Peptide Cm-p5 with Improved Antifungal Activity. ACS Omega.

[18] Høiby N., Ciofu O., Johansen H.K., Song Z.J., Moser C., Jensen P.Ø., Molin S., Givskov M., Tolker-Nielsen T., Bjarnsholt T. (2011). The clinical impact of bacterial biofilms. Int. J. Oral Sci..

[19] Welsh R.M., Bentz M.L., Shams A., Houston H., Lyons A., Rose L.J., Litvintseva A.P. (2017). Survival, persistence, and isolation of the emerging multidrug-resistant pathogenic yeast Candida auris on a plastic health care surface. J. Clin. Microbiol..

[20] Sherry L., Ramage G., Kean R., Borman A., Johnson E.M., Richardson M.D., Rautemaa-Richardson R. (2017). Biofilm-forming capability of highly virulent, multidrug-resistant Candida auris. Emerg. Infect. Dis..

[21] O’Toole G., Kaplan H.B., Kolter R. (2000). Biofilm Formation as Microbial Development. Annu. Rev. Microbiol..

[22] Villar C.C., Kashleva H., Nobile C.J., Mitchell A.P., Dongari-Bagtzoglou A. (2007). Mucosal tissue invasion by Candida albicans is associated with E-cadherin degradation, mediated by transcription factor Rim101p and protease Sap5p. Infect. Immun..

[23] Winter M.B., Salcedo E.C., Lohse M.B., Hartooni N., Gulati M., Sanchez H., Takagi J., Hube B., Andes D.R., Johnson A.D. (2016). Global identification of biofilm-specific proteolysis in Candida albicans. MBio.

[24] Jose A., Coco B.J., Milligan S., Young B., Lappin D.F., Bagg J., Murray C., Ramage G. (2010). Reducing the incidence of denture stomatitis: Are denture cleansers sufficient?. J. Prosthodont..

[25] O’Toole G.A. (2010). Microtiter dish Biofilm formation assay. J. Vis. Exp..

[26] Negri M., Gonçalves V., Silva S., Henriques M., Azeredo J., Oliveira R. (2010). Crystal violet staining to quantity Candida adhesion to epithelial cells. Br. J. Biomed. Sci..

[27] Sherry L., Rajendran R., Lappin D.F., Borghi E., Perdoni F., Falleni M., Tosi D., Smith K., Williams C., Jones B. (2014). Biofilms formed by Candida albicans bloodstream isolates display phenotypic and transcriptional heterogeneity that are associated with resistance and pathogenicity. BMC Microbiol..

